# Bioinformatics Analysis Suggests That SE_1780 Protein From *Staphylococcus Epidermidis* May Be a Member of the Fph Family of Lipases

**DOI:** 10.17912/micropub.biology.001386

**Published:** 2025-02-14

**Authors:** Maya Qaddourah, Sajith Jayasinghe

**Affiliations:** 1 Chemistry and Biochemistry, California State University San Marcos, San Marcos, California, United States

## Abstract

The Protein Data Bank entry for protein SE_1780 from *Staphylococcus epidermidis* lists the function as unknown. We leveraged the framework outlined in the Biochemistry Authentic Scientific Inquiry Laboratory and used bioinformatics tools to ascertain the function of the protein. Based on our analysis, we posit that SE_1780 is a lipase of the α/β hydrolase family with a proposed active site catalytic triad composed of Ser 144, Asp 235, and His 269. Further we identified the lipase FphD as having significant sequence identity to protein SE_1780 and suggest that the protein is a member of the Fph family of lipases from *S. epidermidis* .

**Figure 1. Representation of the structure of protein SE_1780 from S. epidermidis (POI, PDB 3FLE):**
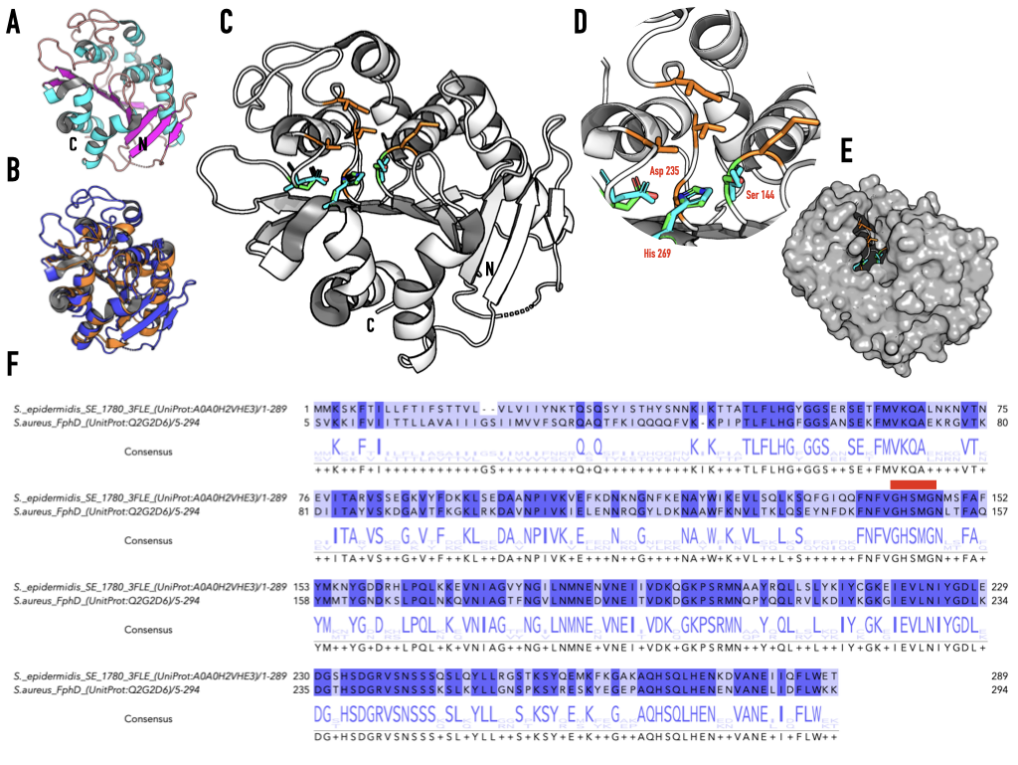
**(A)** N and C indicate the N- and C-termini respectively. The protein contains a central β-sheet composed of eight β-strands, with the leading-edge β-strand running antiparallel to the rest. A total of seven α-helices are distributed on either side of the sheet. **(B)** Comparison of the crystal structure of the POI (blue) with that of *Bacillus subtilis* Lipase A (PDBID: 1I6W, orange). The POI has significant structural similarity to *B. subtilis* Lipase A but differs in two main aspects. The POI is 77 residues longer than *B. subtilis* Lipase A and contains eight β-strands in its core β-sheet compared to six in *B. subtilis* Lipase A. The POI has a much longer loop between β-stands 6 and 7 compared to *B. subtilis* Lipase A. **(C and D)** The proposed catalytic triad of the POI. Based on the location of the catalytic triad of *B. subtilis* Lipase A we propose that the catalytic triad of the POI is comprised of residues Ser 144, Asp 235, and His 269 (panel D). The catalytic site residues of *B. subtilis* Lipase A (Ser 77, Asp 133, and His 156) are shown in teal in panel D. The proposed nucleophilic Ser 144 is located within the sequence GHSMG which conforms to the GXSXG pentapeptide sequence (where X denotes any amino acids) motif observed in lipolytic enzymes. The active site is surface exposed and lined with hydrophobic residues (orange, Met 145, Ala 173, Val 175, Ile 179, and Val 238). **(E)** Surface representation of the POI with active site residues shown in stick representations. **(F)** Pairwise sequence alignment of the POI (UniProt: A0A0H2VHE3) and *S. aureus* FphD (UniProt: Q2G2D6). Alignment is colored based on sequence identity (~ 56%). The catalytic Serine of the POI is located within the sequence GHSMG (red box). Extended data include a PyMol session file (3FLEActiveSiteWith1I6W.pse) that can be used to visualize the active site of the POI as well as the active site of *B. subtilis* Lipase A.

## Description

Protein SE_1780 from *Staphylococcus epidermidis* (the POI) contains 289 residues and is composed of a central β-sheet surrounded by a collection of α-helices (Protein Data Bank (PDB) ID: *3FLE,* Figure 1A ). The β-sheet is composed of eight β-strands, with seven strands arranged parallel to each other, while the remaining strand (β3), located at the leading edge of the sheet, runs anti-parallel to the rest. Of the seven α-helices, five are located on one face of the sheet and two on the other. This core structure is similar to the architecture of enzymes containing the α/β-hydrolase fold (Nardini & Dijkstra, 1999) . The PDB entry for the protein, 3FLE, lists the function as unknown. We leveraged the framework described in the Biochemistry Authentic Scientific Inquiry Laboratory (BASIL) (Koeppe et al., 2023; Roberts et al., 2019) Course Based Undergraduate Research Experience (CURE) and conducted a bioinformatics analysis to hypothesize a possible function for the POI.

*Structural Similarity Suggests that Protein SE_1780 from Staphylococcus epidermidis is a Lipase:*

To gain insights into the function of the SE_1780 protein, we compared the POI to proteins with known structure using the protein structure comparison server DALI (Holm, 2020) . The server identified several PDB entries with Z-scores greater than 4 with sequence identities ranging from 7-30% (Table S1). The result with the highest Z-score (31.5), highest sequence identity (30%), and lowest RMSD (1.7) to the POI was the protein product from gene *lin2722* from *Listeria innocua* (PDB ID: 3DS8). Unfortunately, the PDB lists the function of this protein as unknown and was thus unable to shed light on the function of our POI. However, a literature search found a report of a computational and in vitro analysis suggesting that the protein from gene *lin2722* is an α/β-hydrolase (Sharkawy et al., 2019) . The structure of the POI is also similar to the structures of lipase Lip_vut1 from the goat rumen metagenome (PDB ID 6NKC), *Bacillus subtilis* lipase A (PDB ID: 5CT6), and *Bacillus pumilus* Lipase A (PDB ID: 7R1K) with sequence identities of 24%, 20%, and 20% respectively (Table S1). Many of the other results of the DALI search were PDB entries of *Bacillus subtilis* lipase A crystalized under various conditions (for example PDB IDs 1R50 and 1I6W) but the results also contained entries described as carboxyesterases, arylesterases, and phospholipases. Thus, the DALI search suggests the possibility that our POI is a lipase in the α/β hydrolase family.

*The POI may be a homolog of S. aureus FphD:*

A BLAST search against the proteins in the Esterase and α/β-hydrolase enzymes and relatives (ESTHER) database (Lenfant et al., 2012) indicated that the POI has a 56% sequence identity to the *Staphylococcus aureus* cell surface protein FphD. FphD has yet to be functionally and structurally characterized but is described as belonging to a group of ten α/β-hydrolase enzymes known as fluorophosphonate-binding hydrolases (Fphs) which are produced during biofilm formation (Fellner, 2021; Fellner et al., 2020; Lentz et al., 2018) . Three other members of this family of proteins, FphB, FphF, and FphH have been functionally characterized as lipases that cleave lipid ester substrates, and FphF and FphH have been structurally characterized (Fellner, 2021; Fellner et al., 2023) .

*The Catalytic Triad of Protein SE_1780 is comprised of Ser 144, Asp 235, and His 269:*

To ascertain the nature of the active site, we compared the structure of our POI with that of *B. subtilis* lipase A ( Figure 1B ) (Pouderoyen et al., 2001). The POI exhibits significant structural similarity with *B. subtilis* Lipase A (rmsd 2.39). We selected the *B. subtilis* lipase A instead of any of the Fph proteins based on the lower rmsd observed between *subtilis* Lipase A and the POI. The main structural differences between *B. subtilis* lipase A and the POI are in the β-sheet and the loop between β-strands six and seven. *B. subtilis* lipase A is 77 residues shorter than the POI, and its core β-sheet contains only six strands, as opposed to the eight in the POI. *B. subtilis* lipase A also has a much shorter loop between β-strands six and seven compared to the POI ( Figure 1B ).

The region of the POI that corresponds to the location of the catalytic triad residues (Ser 77, Asp 133, and His 156, Figure 1, panel D) of *B. subtilis* show significant structural similarity and therefore we identified the putative catalytic triad of the POI as Ser 144, Asp 235, and His 269 ( Figure 1C and D). The proposed nucleophilic Ser 144 is located on the loop between strands β5 and β6, Asp 235 is located at the end of strand β7, and His 269 is situated in the loop after strand β8. The catalytic serine is found within the sequence GHSMG which conforms to the conserved GXSXG pentapeptide sequence motif found in lipolytic enzymes (Kovacic et al., 2019) . Based on the analysis of diverse lipases, it has been suggested that variations in the conserved pentapeptide sequence may be used to categorize lipases into 19 families. The specific pentapeptide sequence, GHSMG, present in the POI has not been previously identified (Kovacic et al., 2019) . The same pentapeptide sequence is present in FphD but not in the other Fph proteins.

Given the hydrophobic nature of their substrates, lipases have active site cavities lined with hydrophobic side chains. We find that the catalytic triad of the POI is surrounded by several hydrophobic residues: Met 145, Ala 173, Val 175, Ile 179, and Val 238 ( Figure 1D ). In many lipases the hydrophobic active site is covered by a ‘lid’, composed of an amphipathic helix, that protects the active site (Khan et al., 2017) . The active site of the POI is surface exposed and is not covered by a lid region ( Figure 1E ), and in this regard is similar to lipases, such as *B. subtilis* lipase A, that also do not have a lid.

Using the BASIL CURE framework, we postulate that protein SE_1780 from *Staphylococcus epidermidis* identified in PDB entry 3FLE is a lipase and that the protein may be a member of the recently identified fluorophosphonate-binding hydrolases. We are in the process of expressing and purifying the protein to validate our hypothesis.

## Methods

Structural comparison of the POI with proteins in the PDB were conducted using the DALI server (http://ekhidna2.biocenter.helsinki.fi/dali/). DALI results with the highest Z-scores were used as structural homologs. BLAST search was carried out using the NCBI BLAST server (https://blast.ncbi.nlm.nih.gov/Blast.cgi) and pairwise sequence alignments were carried out using EMBOSS Needle (https://www.ebi.ac.uk/jdispatcher/psa/emboss_needle). Molecular visualization and graphics were created using PyMol (The PyMOL Molecular Graphics System, Version 3.0 Schrödinger, LLC).

## Data Availability

Description: Structural similarity of POI to proteins of known structure as determined by the DALI server.. Resource Type: Dataset. DOI: https://doi.org/10.22002/b32ha-1c454 Description: PyMol session file containing the overlay of PDB structures for the POI (3FLE) and B. subtilis Lipase A (1I6W).. Resource Type: Model. DOI: https://doi.org/10.22002/q3vcq-6a495

## References

[R1] Fellner Matthias (2021). Newly discovered Staphylococcus aureus serine hydrolase probe and drug targets. ADMET and DMPK.

[R2] Fellner Matthias, Lentz Christian S., Jamieson Sam A., Brewster Jodi L., Chen Linhai, Bogyo Matthew, Mace Peter D. (2020). Structural Basis for the Inhibitor and Substrate Specificity of the Unique Fph Serine Hydrolases of
*Staphylococcus aureus*. ACS Infectious Diseases.

[R3] Fellner Matthias, Walsh Annabel, Dela Ahator Stephen, Aftab Nadia, Sutherland Ben, Tan Eng W., Bakker Alexander T., Martin Nathaniel I., van der Stelt Mario, Lentz Christian S. (2023). Biochemical and Cellular Characterization of the Function of Fluorophosphonate-Binding Hydrolase H (FphH) in
*Staphylococcus aureus*
Support a Role in Bacterial Stress Response. ACS Infectious Diseases.

[R4] Holm Liisa (2020). Using Dali for Protein Structure Comparison. Methods in Molecular Biology.

[R5] Khan Faez Iqbal, Lan Dongming, Durrani Rabia, Huan Weiqian, Zhao Zexin, Wang Yonghua (2017). The Lid Domain in Lipases: Structural and Functional Determinant of Enzymatic Properties. Frontiers in Bioengineering and Biotechnology.

[R6] Koeppe Julia R., Roberts Rebecca, Hall Bonnie L., Craig Paul A. (2023). The BASIL cure: Using structure to predict function in protein biochemistry. Biophysical Journal.

[R7] Kovacic Filip, Babic Nikolina, Krauss Ulrich, Jaeger Karl-Erich (2019). Classification of Lipolytic Enzymes from Bacteria. Aerobic Utilization of Hydrocarbons, Oils, and Lipids.

[R8] Lenfant Nicolas, Hotelier Thierry, Velluet Eric, Bourne Yves, Marchot Pascale, Chatonnet Arnaud (2012). ESTHER, the database of the α/β-hydrolase fold superfamily of proteins: tools to explore diversity of functions. Nucleic Acids Research.

[R9] Lentz Christian S., Sheldon Jessica R., Crawford Lisa A., Cooper Rachel, Garland Megan, Amieva Manuel R., Weerapana Eranthie, Skaar Eric P., Bogyo Matthew (2018). Identification of a S. aureus virulence factor by activity-based protein profiling (ABPP). Nature Chemical Biology.

[R10] Nardini Marco, Dijkstra Bauke W (1999). α/β Hydrolase fold enzymes: the family keeps growing. Current Opinion in Structural Biology.

[R11] Osipiuk J., Hatzos C., Clancy S., Kim Y., Joachimiak A., Midwest Center for Structural Genomics (MCSG) (2009). SE_1780 protein of unknown function from Staphylococcus epidermidis..

[R12] Roberts Rebecca, Hall Bonnie, Daubner Colette, Goodman Anya, Pikaart Michael, Sikora Arthur, Craig Paul (2019). Flexible Implementation of the BASIL CURE. Biochemistry and Molecular Biology Education.

[R13] Sharkawy Mary, Carter Andrea A., Craig Paul (2019). Function Identification of the Protein Product of Gene Lin2722 from Listeria innocua using Computational and In-Vitro Techniques. Biophysical Journal.

[R14] van Pouderoyen G., Eggert T., Jaeger K.-E., Dijkstra B.W. (2001). THE CRYSTAL STRUCTURE OF BACILLUS SUBTILIS LIPASE: A MINIMAL ALPHA/BETA HYDROLASE ENZYME.

